# An educational program for decreasing catheter-related bloodstream infections in intensive care units: a pre- and post-intervention observational study

**DOI:** 10.1186/s40981-017-0095-4

**Published:** 2017-05-08

**Authors:** Yuichiro Shimoyama, Osamu Umegaki, Tomoyuki Agui, Noriko Kadono, Nobuyasu Komasawa, Toshiaki Minami

**Affiliations:** 1 Department of Anesthesiology, Osaka Medical College, 2-7 Daigaku-machi, Takatsuki, Osaka 569-8686 Japan; 2 Department of Surgery, Osaka Medical College, Takatsuki, Japan

**Keywords:** Educational program, Catheter-related bloodstream infection, Intensive care unit

## Abstract

**Background:**

Central venous catheters (CVCs) are commonly used in the management of critically ill patients. This study aimed to determine whether an educational program could reduce the rate of catheter-related bloodstream infections (CRBSIs) in intensive care units (ICUs).

**Findings:**

All patients admitted to a medical ICU at a college affiliated with the Japan Society of Intensive Care Medicine between January 2008 and December 2014 were surveyed prospectively for the development of CRBSIs. A mandatory educational program (the intervention) targeting an infection control committee consisting of physicians was developed by a multidisciplinary task force to highlight correct practices for preventing CRBSIs. The program included a 30-min video-based introduction, 120-min lectures with a number of hands-on training sessions, a post-test, posters, safety check sheets, and feedback from the infection control committee. Lectures based on the education program were held every 3 months, and participants were free to choose when they attended the lectures. Each participant was required to view the 30-min introduction before attending the 120-min lectures and complete the post-test after each lecture. Safety check sheets were made to ascertain adherence to contents of the educational program. Posters describing the educational program were posted throughout the ICU. A pre- and post-intervention observational study design was employed, with the main outcome measure being yearly CRBSIs. We also calculated cost savings that resulted from improved CRBSI rates.

During the 12-month pre-intervention period, four episodes of CRBSIs occurred in 1171 patient ICU-days (i.e., 3.4 per 1000 patient ICU-days). In the first year after the intervention, the rate of CRBSIs decreased to 0 in 1157 patient ICU-days (*P* ≤ 0.05). The estimated cost savings secondary to this decreased rate for the 1 year following introduction of the program was between 1850,000 and 27,000,000 yen ($14,800–$216,000).

**Conclusions:**

A program aimed at educating healthcare providers on the prevention of CRBSIs led to a dramatic decrease in the rate of primary bloodstream infections. This suggests that educational programs may substantially decrease medical care costs and patient morbidity attributed to central venous catheterization when implemented as part of mandatory training.

## Findings

### Background

Central venous catheters (CVCs) are commonly used in the management of critically ill patients. In the USA, 15 million CVC days (i.e., the total number of days of exposure to CVCs among all patients in a selected population during a selected time period) occur in intensive care units (ICUs) each year [1]. According to data regularly reported by the National Nosocomial Infection Surveillance (NNIS) system in the United States, more than 85% of primary bacteremias are catheter-related [2–4]. Catheter-related bloodstream infections (CRBSIs) cause considerable morbidity and mortality, and lead to high healthcare costs. Mortality attributable to CRBSIs has been estimated to be as high as 35%, and length of hospital stay is consistently increased among infected patients in published reports [5, 6]. Each year in the USA, CVCs cause an estimated 80,000 CRBSIs and, as a result, up to 28,000 deaths among patients in ICUs. These infections are associated with up to $2.3 billion in annual costs [7].

To improve patient outcomes and reduce healthcare costs, there is considerable interest in reducing the incidence of these infections. Although there is no consensus on the optimal approach to achieve this, several recent studies indicate that educating healthcare providers on evidence-based approaches to prevent these infections can decrease infection rates [8–11]. The Centers for Disease Control and Prevention (CDC) recommends healthcare provider education as an important element of programs aimed at preventing hospital-acquired infections [12].

Against this backdrop, we carried out a clinical investigation to determine whether an educational program could reduce the rate of CRBSIs in the ICU of a teaching hospital. This study aimed to evaluate the effects of the program for up to 48 months after its implementation.

## Methods

### Study location and patients

This study was judged to not require registration by the institutional review board of Osaka Medical College (Osaka Japan) due to the use of unlinked anonymous data. This study was conducted in a medical ICU at a college affiliated with the Japan Society of Intensive Care Medicine. The medical ICU (8 beds) is an open unit with a multidisciplinary team providing patient care under the direction of attending physicians who are board certified in critical care medicine. Nurse staffing is maintained at a ratio of two patients per nurse. From January 2008, all patients admitted to the medical ICU were surveyed for the occurrence of CRBSIs. Intravascular catheters (e.g., CVCs, dialysis catheters, pulmonary artery catheters) used in the hospital were standard catheters without antimicrobial or antiseptic coatings. Arterial catheters were not surveyed in this study. The use of ultrasound/Doppler is recommended during insertions, except in emergent cases. Mostly residents (3–6 years of experience) and fellows (7–12 years of experience) insert CVCs at our hospital, and are required to use maximal sterile barrier precautions, including the use of a cap, mask, sterile gown, sterile gloves, and a sterile full body drape, when inserting CVCs. The primary study hypothesis was that the rate of CRBSIs would be reduced during the first year after implementation of the educational program (intervention) as compared with baseline. The secondary hypothesis was that the observed decrease in infection rate between 0 and 12 months after implementation of the program would be sustained over the subsequent observation period.

### Study design and data collection

A pre- and post-intervention observational study design was employed with the main outcome measure being yearly CRBSIs. The educational program was developed in 2010 by an infection control committee comprising a physician, surgeon, anesthesiologist, intensivist, and nurses. The 2009 calendar year served as the baseline period for the incidence of CRBSIs. Throughout the study, data on the number of CRBSIs, as well as the following parameters, were recorded prospectively and on a daily basis: occurrence of primary bacteremia, number of patient ICU-days prior to the onset of bacteremia, and the species of microorganism associated with bloodstream infection. To coincide with the implementation period of the study intervention, daily data were aggregated into 1-year periods. The yearly rate of infection was calculated as the number of infections per 1000 patient-ICU days for each 1-year period. Yearly rates were assigned to one of six categories based on when the study intervention was implemented: at baseline, during the implementation period, or during one of four 1-year intervals up to four years after implementation. Data on who inserted the central catheters, average duration of catheter use, and number of lines in individual patients were not collected.

### Definitions

A central catheter is defined herein as a catheter that ends at or near the heart or in a great vessel near the heart, and includes peripherally-inserted central catheters. CRBSIs were classified as primary or secondary based on CDC NNIS definitions [13]. Primary bloodstream infection (bacteremia) was defined using either of the following two criteria: (1) isolation of a recognized pathogen from blood culture (*Staphylococcus aureus*, *Enterococcus* species, *Candida* species) unrelated to infection at another site, and (2) fever of ≥38.0 °C, chills, or hypotension, and either of the following: common skin contaminant (e.g., *Bacillus* species, *Propionibacterium* species, coagulase-negative *staphylococci*, or *micrococci*) isolated from two blood cultures drawn on separate occasions within 24 h that are unrelated to infection at another site, or a common skin contaminant isolated from a blood culture of a patient with an intravascular device and the physician institutes appropriate antimicrobial therapy. CRBSI was defined as primary bacteremia in the presence of a CVC. Secondary bacteremia was defined as a bloodstream infection that develops as a result of a documented infection with the same microorganism at another body site.

### Intervention/educational program

Newly hired physicians (interns, residents, fellows, attending physicians) were required to complete the educational program, which covered indications for intravascular catheter use, proper procedures for the insertion and maintenance of intravascular catheters, and appropriate infection control measures to prevent intravascular catheter-related infections. The program was implemented from April 2010 and consisted of a 30-min video introduction, 120-min lectures with a number of hands-on training sessions (not required for fellows and attending physicians given their experience), a post-test (not required for attending physicians given their experience), posters, safety check sheets, and feedback from the infection control committee. Lectures based on the education program were held every 3 months, and participants were free to choose when they attended the lectures. Contents of the safety check sheets were entered through each patient’s computer terminal located directly outside the patient’s room after inserting the CVC to improve clinician-to-medical safety committee communication within the hospital. This process allowed for monitoring adherence to the program’s contents. The 120-min lectures and post-test were held every three months and based on CDC guidelines. The post-test was held after the 120-min lectures to reinforce the topic and group discussion of hands-on training. A score of 85% correct was required for the post-test. If this score was not achieved, the post-test was repeated until a passing score was achieved. Topics covered in the 120-min lectures and post-test included the epidemiology of CRBSIs, aseptic technique, use of maximal barrier precautions during central vein catheter insertion, preference for the jugular vein as a central vein insertion site, routine central vein catheter site care, proper technique for obtaining blood cultures, and guidelines for changing IV tubing and administration sets. The program was revised and recently updated in 2013 (Table 1). However, the updates were minor and the contents had not substantially changed since 2010. Furthermore, item 1: “Use a subclavian site, rather than a jugular or a femoral site” is the general rule of our hospital. Limited to using in the operation room and ICU, it is stated clearly that a jugular site is the first choice by consideration of possibility of receiving positive pressure ventilation for patients. In addition to the educational program, a promotional campaign was launched beginning in April 2010 to augment the educational program. The campaign involved providing stickers on ID cards of staff members who completed the educational program, as well as posters describing the program which were displayed throughout the hospital.

**Table 1:** Hospital guidelines for the prevention of catheter-associated bloodstream infection

1.	Use a subclavian site, rather than a jugular or a femoral site, in adult patients to minimize infection risk for nontunneled CVC placement.
2.	Avoid using the femoral vein for central venous access in adult patients.
3.	Use a CVC with the minimum number of ports or lumens essential for the management of the patient.
4.	Promptly remove any intravascular catheter that is no longer essential.
5.	Perform hand hygiene procedures, either by washing hands with conventional soap and water or with alcohol-based hand rubs (ABHR). Hand hygiene should be performed before and after palpating catheter insertion sites as well as before and after inserting, replacing, accessing, repairing, or dressing an intravascular catheter.
6.	Maintain aseptic technique for the insertion and care of intravascular catheters.
7.	Use maximal sterile barrier precautions, including the use of a cap, mask, sterile gown, sterile gloves, and a sterile full body drape, for the insertion of CVCs, PICCs, or guidewire exchange.
8.	Prepare clean skin with a >0.5% chlorhexidine preparation with alcohol before central venous catheter insertion and during dressing changes.
9.	Use either sterile gauze or sterile, transparent, semipermeable dressing to cover the catheter site.
10.	Use a sterile sleeve for all pulmonary artery catheters.
11.	Do not administer systemic antimicrobial prophylaxis routinely before insertion or during use of an intravascular catheter to prevent catheter colonization or CRBSI.
12.	Do not routinely replace CVCs, PICCs, hemodialysis catheters, or pulmonary artery catheters to prevent catheter-related infections.

*Abbreviation*: *CVC* central venous catheter, *PICC* peripherally inserted central catheter, *CRBSI* catheter-related bloodstream infection

### Blood culture technique

Blood samples were obtained from two peripheral sites by physicians. Before collecting the blood sample, the skin was disinfected with 70% isopropyl alcohol and followed by 2% iodine tincture. When only one peripheral site was available and the patient had a central vein catheter in place, the second blood culture sample was obtained from the CVC [14]. Blood samples from CVCs were obtained from needleless caps that were disinfected with 70% isopropyl alcohol, allowed to dry, and wiped with a povidone-iodine pad for 30 s. Excess povidone-iodine was wiped off with sterile gauze prior to obtaining the sample. Three milliliters of blood were aspirated and discarded from both the CVC and peripheral venipuncture. A new syringe was used to aspirate an additional 20 mL of blood. A blood volume of 10 mL was injected into each of the two blood culture bottles. Injection of ≤5 mL of blood into a blood culture bottle was prevented in order to avoid false-negative results [15]. Attending physicians at the medical ICU and infection control staff at the hospital adjudicated contaminated cultures before submitting data.

### Cost analysis

Numerous studies have attempted to quantify the costs associated with CRBSIs. The lowest of the published estimates comes from Arnow et al. [16], who estimated the cost to be $3700 per infection using 1991 dollars, upon examining CVCs, arterial lines, and peripheral IV catheters in both floor and ICU patients. In more recent analyses, estimated costs associated with CRBSIs vary, ranging from $11,971 to $54,000 per episode of infection [17, 18]. We calculated cost savings based on these previous studies in order to determine the impact of improved CRBSI rates resulting from the education program.

### Statistical analysis

Statistical analysis was performed using SPSS 17.0 for Windows (SPSS Inc., Chicago, IL, USA). Fisher’s exact test was used to compare categorical variables. The incidence rate of CRBSIs per 1000 patient ICU-days was calculated, and the risk difference of CRBSIs in the post- vs pre-intervention period was determined. All reported *P* values are two-sided. *P* ≤ 0.05 was considered statistically significant.

## Results

During the 12-month pre-intervention period (calendar year 2009), a total of four episodes of CRBSI occurred during a total of 1171 patient ICU-days. This amounts to an infection rate of 3.4 per 1000 patient ICU-days. During the post-intervention period (calendar year 2011), no episodes of CRBSI were recorded for a total of 1157 patient ICU-days. The rate of CRBSIs decreased from 3.4 infections per 1000 patient ICU-days at baseline to 0 at 0–12 months after implementation of the program (*P* ≤ 0.05). The difference in CRBSI rates between the pre- and post-intervention periods was 3.4 per 1000 catheter ICU-days. However, the markedly reduced rates of CRBSI were not sustained for up to 36 months of follow-up (Table 2).

**Table 2:** Baseline data

Study period (year)	N	No. of infections	Patient ICU-days	No. of infections per 1000 patient ICU-days	Incidence-rate ratio	*P* value
Baseline (2009)	176	4	1171	3.4	1	
Begin implementation (2010)	172	0	1189	0	-	
After implementation						
1 year (2011)	195	0^※^	1157	0	-	0.0497
2 years (2012)	191	1	1722	0.58	0.11	0.1985
3 years (2013)	171	1	1438	0.7	0.14	0.3716
4 years (2014)	207	1	1598	0.68	0.13	0.1845

^※^ *P* value < 0.05 was considered statistically significant

Abbreviation; *ICU* intensive care unit

### Cost analysis

Assuming a continued infection rate of 3.4 per 1000 patient ICU-days (pre-intervention rate), four infections would have been expected to occur for the 1157 patient ICU-days in the 12 months after the intervention. When the reduced number of CRBSI occurrences (total of four) resulting from the educational program was calculated, the savings associated with improvements ranged from ¥1,850,000 to ¥27,000,000 ($14,800–$216,000) annually using the yen-dollar exchange rate in 2015.

## Discussion

This study demonstrated that an educational program for physicians working at Osaka Medical College was able to significantly reduce the incidence of CRBSIs. These results suggest that behavioral changes may have played a crucial role in the success of the program. Simulation-based learning was recently found to be more effective than video training alone in improving resident skills [19]. In the context of the present study, simulation-based learning substantially reduced the CRBSI rate relative to the control group. Overall, our findings suggest that an education-based intervention aimed at optimizing CVC care could be successfully implemented in a teaching hospital and have significant public health consequences. Importantly, the intervention can be implemented without the need for expensive technology or additional ICU staffing.

Our results are consistent with those of published studies reporting significant reductions in the occurrence of hospital-associated infections with the implementation of education-based interventions. For instance, previous studies have reported 66, 58.5, and 27% reductions in the incidence of CRBSIs as a result of implementing education-based programs [8, 20, 21]. Education-based programs have previously reduced ventilator-associated pneumonia (57.6% decrease in infections in five ICUs) [10]. Use of the same educational program in a pediatric hospital and a community hospital had similar results [11]. In one study, an educational program was associated with a 53.6% reduction in patient colonization of vancomycin-resistant *enterococci* [22]. These results and published studies support the following recommendations of the CDC: “Educate healthcare personnel regarding the indications for intravascular catheter use, proper procedures for the insertion and maintenance of intravascular catheters, and appropriate infection control measures to prevent intravascular catheter-related infections” (Category IA of the Guidelines for the Prevention of Intravascular Catheter-related Infections, 2011 [23]).

Within a year after implementation, the rate of CRBSIs was reduced to 0. However, this significant reduction was not sustained over a longer period (i.e., throughout 3 years of follow-up; Table 2). This result suggests that beneficial effects of the intervention may not persist for long periods. What can we do to maintain the beneficial effects over a longer period? An important common element underlying the success of the program was the wide exposure of the program to hospital staff who directly care for patients consecutively. The effects of these educational programs may be even more pronounced in the longer-term if these hospital staff are involved in designing the specific measures taught in the program. In the context of this study, the educational program targeted the infection control committee, which consisted of physicians. This should be purposefully done in order to impact healthcare providers most closely involved in performing the interventions described in the program.

CRBSI rates greater than 1 or 2 per 1000 patient ICU-days are no longer acceptable in current medical settings [24]. The consistent impact of educational programs on the reduction of CRBSIs suggests that their implementation should be routine in hospital environments where patients are at risk of being infected. Efforts to increase compliance with the established protocols should be discussed and updated on a regular basis, and continuous surveillance of CVC infection rates with feedback to staff should be implemented. We suggest that educational programs be renewed/updated in intervals of several years and making participation in the programs mandatory for all new employees.

Despite our positive outcomes, there are a number of limitations worth noting. First, the study was conducted in an ICU of a single hospital, so the results may not be broadly generalizable. Second, we did not collect data based on the positions of hospital staff (e.g., interns, residents, fellows, attending physicians) who inserted the central catheters, or the varying experiences of attending physicians versus interns, for example, regarding CVC insertion. This raises the possibility that differences in behavior/experience other than the implementation of the educational program may have accounted for these results. Third, we did not record the duration of central venous catheterization or the number of lines in individual patients. An unrecognized reduction in the duration of catheterization or the number of lines could have influenced our results. Fourth, our study design does not allow us to determine which aspects of the intervention accounted for the reduction in the rate of CRBSIs. Another potential limitation is that we did not evaluate outcomes other than bloodstream infection. As a result, we cannot determine whether the intervention influenced antibiotic utilization, length of hospital stay, mortality, or antibiotic resistance patterns.

Despite these limitations, our results indicate that implementation of the educational program directly or indirectly reduced the rate of CRBSIs. Thus, we were able to achieve the original aim of the study, despite the limitations discussed above and inability to identify the specific aspects of the intervention accounting for the reduction.

## Conclusions

Here, we demonstrated that a relatively simple educational program can reduce CRBSIs in a hospital setting. The observed reduction in rate from 3.4 per 1000 patient-ICU days to 0 per 1,000 patient-ICU days was associated with an estimated cost savings of between ¥1,850,000 and ¥27,000,000 annually within the first year after implementing the program. However, rates did not remain significantly reduced throughout the remaining three years of follow-up. Although the program needs further refinement, our findings suggest that the implementation of education-based infection control programs is important for the optimal functioning of medical ICUs.

## Abbreviations

CDC: The Centers for Disease Control and Prevention
CRBSI: Catheter-related bloodstream infection
CVC: Central venous catheter
ICU: Intensive care units
NNIS: National Nosocomial Infection Surveillance
